# Thermoelectric Responsive Shape Memory Graphene/Hydro-Epoxy Composites for Actuators

**DOI:** 10.3390/mi7080145

**Published:** 2016-08-22

**Authors:** Yongkun Wang, Wenchao Tian, Jianqiang Xie, Yan Liu

**Affiliations:** 1 Key Laboratory of Ministry of Education for Electronic Equipment Structure Design, Xidian University, Xi’an 710071, China; wctian@xidian.edu.cn (W.T.); liuy@xidian.edu.cn (Y.L.); 2 Department of Polymer materials and Engineering, College of Material Science and Engineering, North China University of Science and Technology, Tangshan 063009, China; xiejianqiang@ncst.edu.cn; 3 State Key Laboratory for Manufacturing Systems Engineering, Xi’an Jiaotong University, Xi’an 710054, China

**Keywords:** shape memory polymer, graphene, shape memory behavior, actuation materials

## Abstract

A series of thermoelectric responsive shape memory hydro-epoxy (H-EP) composites filled with different contents of graphene were developed and characterized. Compared with traditional actuation materials, these novel shape memory composites exhibit attractive properties, such as light weight, large deformation, good processability and high response speed, making them good candidates for actuator materials. The effect of graphene content on the shape memory composites was studied in terms of mechanical, dynamic mechanical analysis (DMA), electrical properties, and thermoelectric responsive shape memory test. The results show that when graphene content was 2 wt %, the bend strength of the composite improved by about 47% with a storage modulus larger than other composites. The shape recovery ratio of the composites was about 100%, and the shape recovery speed increased with the increment of graphene content, applied voltage, and temperature. Due to the excellent actuation performance, the graphene/hydro-epoxy composite has potential applications in the actuator in the future.

## 1. Introduction

Actuators are mechanical devices that are used to change or control mechanisms or systems, which are operated by responding to appropriate external stimulus, such as heat, electric, light, magnetic, pH, specific ions, and pneumatic [1]. Actuators have been widely used in micropump, switches, medical devices, and many other smart structures [2,3]. Until recently, the most commonly used materials for actuation materials are shape memory materials (SMM). SMMs are able to perceive and respond to the stimulation of environmental changes, and the mechanical parameters (such as shape, position, strain, etc.) can be adjusted to restore it to its original state. SMMs include shape memory alloy (SMA), shape memory ceramic (SMC), and shape memory polymer (SMP) [4,5,6].

Since 1984, SMPs have been known for the attractive properties, such as light weight, large deformations (strain of about 300%–400%), and being easily formable into arbitrary shapes compared to SMA and SMC [7,8,9]. Many types of SMPs have been prepared, including thermosetting SMPs, thermoplastic SMPs, and shape memory blends. However, there are still many problems that restrict the application of these SMPs in the actuator. One is that the shape recovery force of SMPs is not large. Another important problem is that the stimulation for the SMPs is single. Therefore, the shape memory polymer composites in this study were developed. However, another problem for the shape memory polymer composites used in the actuator is that the external force is not easy to obtain [10,11,12]. In response to these limitations, novel shape memory polymer composites that can sense more than one stimulus, have large shape recovery force, and rapid response were prepared in this study.

In this paper, graphene was used to fill the shape memory hydro-epoxy (H-EP) to prepare novel thermoelectric responsive shape memory polymer composites. Graphene is a stable two-dimensional single-carbon atom layer crystal material. It has a large number of excellent properties, such as excellent electrical (10^−8^ Ω/m) and thermal conductivity (5000 Wm^−1^·K^−1^), high surface area, excellent mechanical strength (modulus, 1100 GPa), and high flexibility. Due to these properties, graphene can be used to improve the properties of the SMPs and render the shape memory polymer composites to be a possible competitive candidate for actuation materials [13,14,15]. Therefore, there are many reports about the SMPs filled with graphene. Yoonessi et al. [16] doped polyimide with graphene to prepare shape memory nanocomposites. The results show that the graphene improved the mechanical performance, recovery rate, and thermal stability of the nanocomposite. Kim et al. [17] investigated the electro-active shape memory behavior of graphene/polyurethane nanocomposites. They found that the shape fixity ratio of the composites was not changed and shape recovery ratio slightly decreased with additional graphene. However, there have been no report on the MEMS actuator materials-based shape memory graphene/H-EP composites. Therefore, our objective is to prepare a novel shape memory polymer composites for actuator and investigate the performance of the thermal and electrical stimulated actuation.

## 2. Experimental

### 2.1. Materials and Preparation

The H-EP (AL3040, epoxy value is 0.43 eq/100 g) was produced by Yantai Aolifu Chemical Industry Co., Ltd. (Yantai, China). The curing agent methyl tetrahydro-phthalic anhydride (MeTHPA, molecular weight = 166.181 Da) was purchased from Shanghai Western Science and Technology Co., Ltd. (Shanghai, China). The accelerant agent 2,4,6-tris(dimethylaminomethyl) phenol (DMP-30, Molecular weight is 265.4 Da) was provided by Guangzhou Kay Trade Co., Ltd. (Guangzhou, China). High purity graphene (purity > 98 wt %) was obtained from Chengdu Organic Chemistry Co., Ltd. (Chengdu, China). *N*,*N*-dimethylformamide (DMF) was supplied by SigmaAldrich (Shanghai, China). All of them were used without further purification.

### 2.2. Sample Preparation

The samples were fabricated by incorporating different contents of high purity graphene (1, 2, and 3 wt %) into H-EP resin. Stable dispersions of graphene were created by ultrasonic dispersion (100 W) for 1 h in DMF. A certain mass ratio of the H-EP, MeTHPA, and DMP-30 (the weight ratio is H-EP:MeTHPA:DMP-30 = 100:80:0.8) were mixed at 60 °C, the mixture was stirred by magnetic stirrer until the mixture was evenly mixed. Then, the graphene/DMF solution was added to mixtures stirred by a high-speed magnetic stirrer at a speed of 2000 rpm/min for 10 min, and the mixtures were sonicated by an ultrasonic dispersion instrument for 20 min. The mixture was heated to 60 °C in an oil bath to evaporate the solvent and the magnetic stirring speed is controlled to 300 rpm/min for 2 h. After that, the mixture was placed in a vacuum oven at a temperature of 80 °C for 10 h. Finally, the mixture was poured into a glass mold for curing and the curing temperature process was 80 °C/2 h + 120 °C/2 h + 150 °C/3 h.

### 2.3. Measurements

#### 2.3.1. Mechanical Properties

The tensile and bending tests were carried out on a universal testing machine (AGS-X, Shimadzu, Kyoto, Japan) according to ASTM D638-14 [18] and ASTM D7264/D7264M-15 [19].

#### 2.3.2. Electrical Properties

The resistance of the graphene/H-EP composites was tested using a modern digital multimeter (UT61E).

The volume resistivity of the samples was calculated by the following formula:

(1) $$\rho =R\frac{A}{L}$$

Where *ρ* is the volume resistivity, *R* is the resistance, *A* is the contact area between the electrode and the composite (1.0 × 0.2 cm^2^), and *L* is the length of the sample.

#### 2.3.3. Scanning Electron Micrsocopy (SEM)

The specimens were frozen in liquid nitrogen, and then brittle fractured. The broken surfaces were sputter-coated with gold and observed with a SEM (INCA X-ACT, Tescan, Brno, Czech republic).

#### 2.3.4. Dynamic Mechanical Analysis (DMA)

The samples were cut into 5 × 10 × 30 mm^3^, and the samples were sanded with 800 grit sandpaper (Shandong lianjie engineering materials Co., LTD, Jinan, China). DMA was performed in a single cantilever clamping fixture and carried out using a Q800 (TA instruments company, NewYork, NY, USA). The temperature was increased to 180 °C from 20 °C with a rate of 5 °C/min, the amplitude and dynamic load were 10 um and 1 Hz, respectively.

#### 2.3.5. Thermo-Active Shape Memory Property

**Figure 1:**
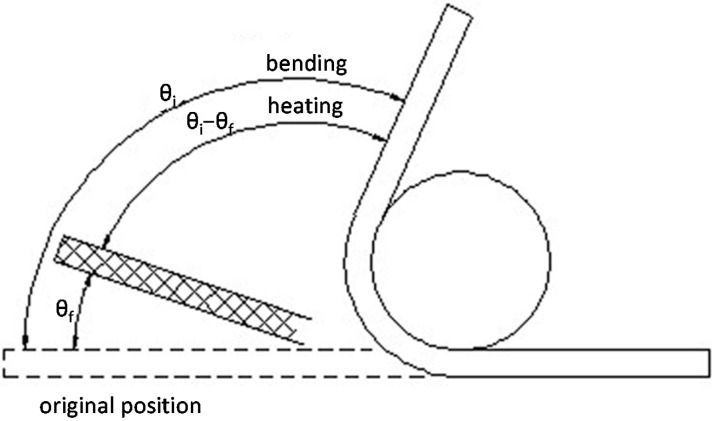
Shape memory model.

Rectangular samples (100 × 10 × 3 mm^3^) were cut to investigate the thermo-active shape memory effect of the graphene/H-EP composites. Shape memory performance of the composites were tested as follows: (i) in the oven, heating the samples to the glass transition temperature (*T*_g_) and held for 5 min; (ii) bending the samples into a “U” shape and then quickly dipping the samples into ice water to maintain the external force; (iii) putting the “U”-shaped samples into hot water and the temperature of the water set to *T*_g_, *T*_g_ + 10 °C, and *T*_g_ + 20 °C of the composites; and (iv) observing the shape recovery process of composites, when the shape of the samples did not change, recording the recovery time. Figure 1 shows the shape memory model. The shape memory recovery speed is defined as *θ_i_/t*, and the shape recovery ratio is defined as (*θ_i_* – *θ_f_*)/*θ_i_* × 100% [8], where, *θ_i_* is the deformation angle (^o^), *t* is the recovery time, and *θ_f_* is the angle of recovery after deformation.

#### 2.3.6. Electroactive Shape Memory Behavior Test

Rectangular samples (100 × 10 × 3 mm^3^) were cut to test the electro-active shape memory behavior under different voltages. The first two steps in the process of the electro-active test were the same as thermo-active shape memory test. However, the third step is different. In the electro-active shape memory test, different voltages were applied to the samples to observe the shape memory process and recorded the shape recovery time.

## 3. Results and Discussions

### 3.1. Mechanical Properties and SEM

**Figure 2:**
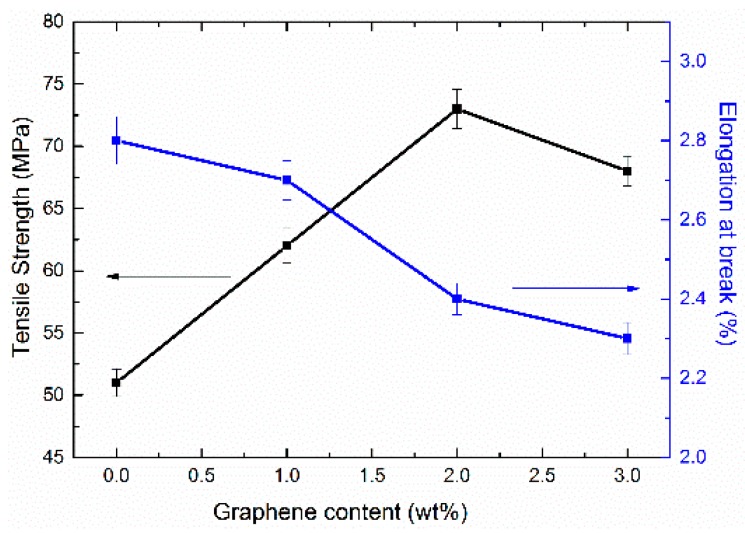
Tensile properties of the graphene/H-EP composites.

The tensile property is one of the most important indicators to reveal the mechanical property of graphene/H-EP composites, and the relationship between mechanical properties and graphene content is illustrated in Figure 2, where each sample is characterized by tensile tests at room temperature. The curves reveal that: (i) the tensile strength of graphene/H-EP composites increases with the increment of graphene, but decreases when the graphene content increases to 3 wt %. Considering the graphene is the reinforcement, the tensile strength of the composites should increase. However, the tensile strength would be offset by the uneven dispersion of reinforcement; (ii) it is of significance that the elongation at break of all specimens decreases with the increase in the content of graphene.

**Figure 3:**
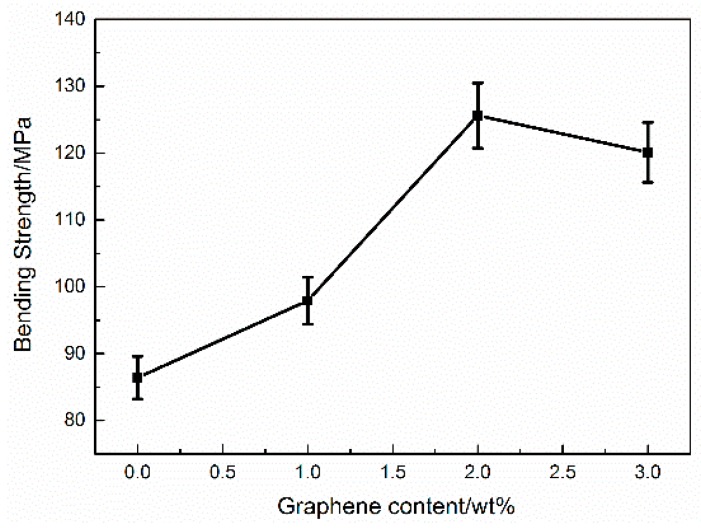
Bending strength of the graphene/H-EP composites (Error bars are standard deviatiion).

**Figure 4:**
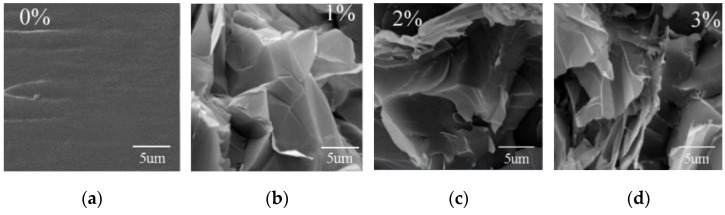
SEM of the graphene/H-EP composites. (**a**) Pure H-EP resin; (**b**) 1 wt % graphene/H-EP; (**c**) 2 wt % graphene/H-EP; (**d**) 3 wt % graphene/H-EP.

The relationship between bending strength and graphene content is shown in Figure 3. The bending strength of the bulk specimen is 86.4 MPa, and the bending strength of sample with 2.0 wt % graphene increases by 45.37% compared to the 0 wt %. Considering the effect of graphene reinforcement, the bending strength of the composites will increase. It is noted that the bending strength of the sample with 3.0 wt % graphene decreases slightly; this reduction is caused by the uneven dispersion of graphene in the matrix. For further analysis, SEM images of the samples were observed. From Figure 4, it can be found that the surface of the bulk sample is smooth, and the graphene is dispersed uniformaly in the H-EP matrix when the graphene content is less than 2.0 wt %. However, when the graphene content increases to 3.0 wt %, the graphene appears to agglomerate and fold, which is due to the high surface energy and van der Waals force of the graphene.

### 3.2. Thermal Properties Analyses

**Figure 5:**
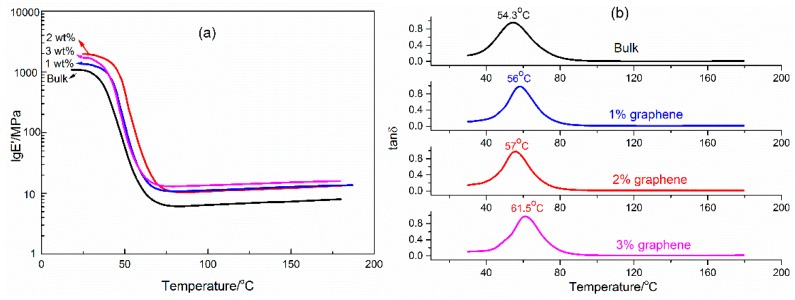
DMA curves of the graphene/H-EP composites: (**a**) storage modulus (lg*E*’) of graphene/H-EP composites; and (**b**) tan*δ* curves of graphene/H-EP composites.

Generally, when the temperature is lower than the shape memory transition temperature, the storage modulus of a good shape memory polymer composite is 2–3 orders higher than that when the temperature is above the shape memory transition temperature [20]. In this paper, the shape memory transition temperature is the glass transition temperature (*T*_g_). Figure 5a indicates that the storage modulus of the graphene/H-EP composites complies with the above rules, which means that the graphene/H-EP composites are good shape memory materials. For example, the storage modulus of the shape memory H-EP composite with 2.0 wt % graphene at 25 °C is 1979.16 MPa, yet it is 11.12 MPa at 110 °C. The storage modulus below shape memory transition temperature is related to the shape fixed ratio of the composites, and the storage modulus above shape memory transition temperature is related to the shape recovery ratio. It also can be found in Figure 5a that the storage modulus increases with the increment of the graphene content but decreases when the graphene content rise to 3.0 wt %. Graphene has high surface energy, enabling the physical crosslinking between graphene and the molecular chain of H-EP [21,22,23]. Therefore, the degree of physical crosslinking gradually increases with increasing graphene content. However, when the graphene content rises to 3.0 wt %, the agglomeration of graphene leads to a decrease in physical crosslinking. Thus, the storage modulus of the graphene/H-EP composites increases first and then decreases.

*T_g_* is an important parameter of themomechanical property and shape memory effect of shape memory polymer composites. In this study, the *T*_g_s of the SMPs are the peaks of the tan*δ* curves, as shown in Figure 5b. The *T*_g_s of the bulk specimens with 1.0, 2.0, and 3.0 wt % graphene are 54.3, 56, 57 and 61.5 °C, respectively. The *T*_g_ increases slight with the increasing of the graphene content, this phenomenon is due to the doped graphene hindering the mobility of the H-EP molecular chain [24].

**Figure 6:**
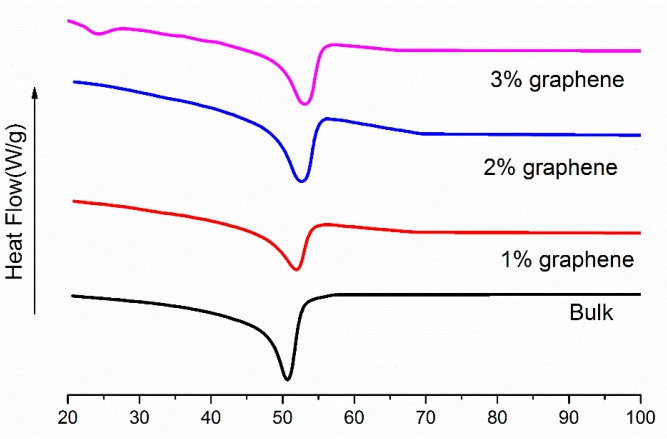
DSC curves of the graphene/H-EP composites.

Figure 6 illustrates that the glass transition temperature of the composites is about 52 °C and the peak moves to a higher temperature with an increase in the graphene content. Note that the graphene as fillers will hinder the movement of molecular chains. This is considered as friction interaction that would help SMP composites to resist external loading, resulting in the improvement of the glass transition temperature.

### 3.3. Electrical Property Analysis

**Figure 7:**
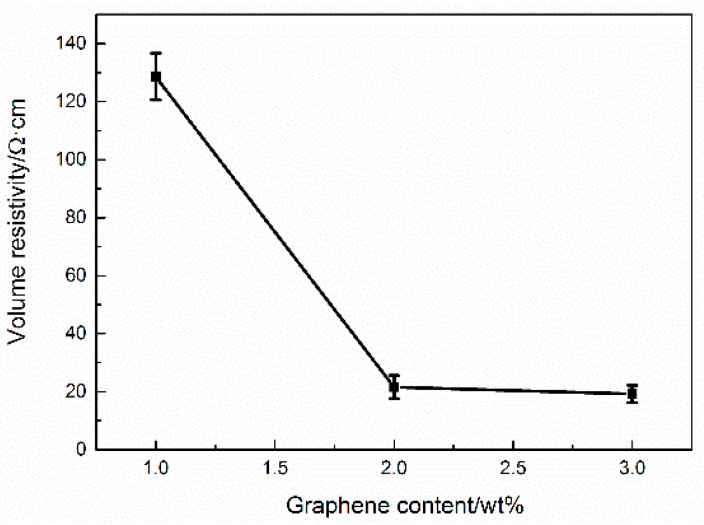
The relationship between volume resistivity and graphene content of the composites (Error bars are standard deviatiion).

Due to the excellent electrical properties of graphene, the volume resistance of graphene/H-EP composites decrease as the graphene content increases, as shown in Figure 7. The electrical conductivity of the composites is related to many factors, the type of fillers, the size of fillers, and the shape of fillers. However, the most important influential factor is the content of the conductive fillers [22]. Figure 7 reveals that when the graphene content increases to 2.0 wt %, the volume resistivity of the composites is about 21 Ω·cm, which means the composite becomes to an excellent conductor.

### 3.4. Thermo-Active Shape Memory Property Analysis

**Figure 8:**
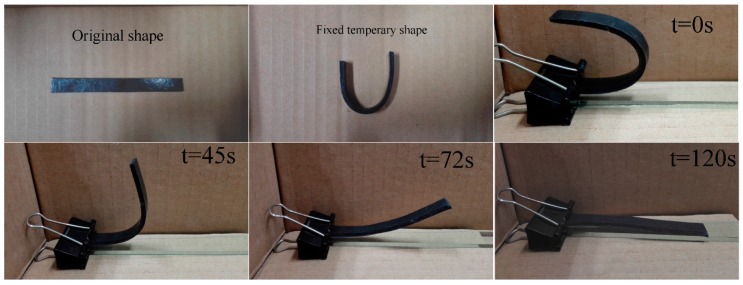
The shape recovery process of the sample with 1.0 wt % graphene in a 55 °C oven.

The thermo-active shape memory property results show that the graphene/H-EP composites can be fully recovered within a few minutes at three different temperatures. This means that the shape recovery ratio of the graphene/H-EP composites are about 100%. This indicates that the graphene/H-EP composites have good shape memory behavior. In addition, it also shows that the addition of small amounts of graphene do not affect the shape memory behavior of graphene/H-EP composites. The shape memory process of sample with 1.0 wt % graphene is shown in Figure 8. The original shape of the sample is a planar rectangular shape, the specimen is heated to the temperature of *T*_g_ and deformed into a temporary U shape. After cooling, the temporary shape is fixed. Then, putting the sample in the oven and raising the temperature to 55 °C, the sample takes 120 s to completely return to its original shape.

**Figure 9:**
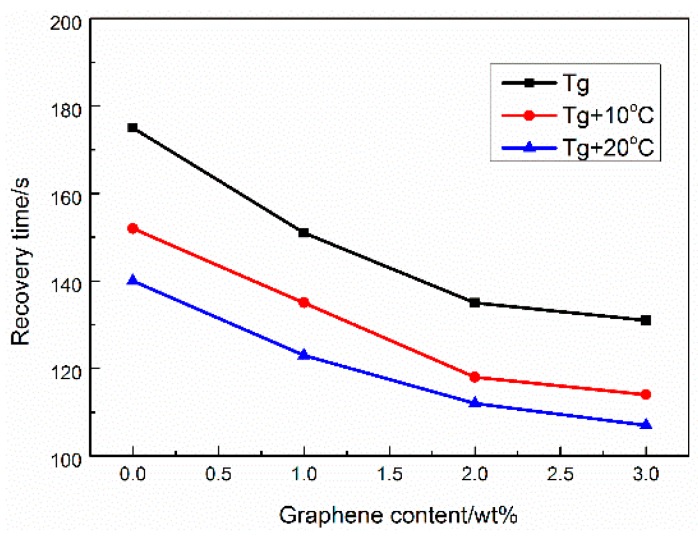
The effect of the temperature on the shape recovery time of the composites.

Figure 9 shows the shape recovery time at *T*_g_, *T*_g_ + 10°C, and *T*_g_ + 20 °C, it can be seen that the shape recovery time decreases with increasing graphene content. During the process of the thermo-active shape memory test, the specimen is deformed at high temperature, and then quickly cooled under the condition of keeping the external force. At this moment, the stress will be frozen as the recovery force. When the sample is heated again, the frozen stress is released, which drives the deformed sample to recover to its original shape. Therefore, the shape recovery speed and shape recovery time are related to the recovery force. The faster the recovery force is released, the faster the shape recovery speed and the lower the shape recovery time [25]. Meanwhile, due to the excellent thermal conductivity of graphene, the composites with more graphene content have higher thermal conductivity, and the composites can reach to the *T_g_* more quickly, which results to the recovery time decreases with the increment of the graphene content.

### 3.5. Electro-Active Shape Memory Property Analysis

**Figure 10:**
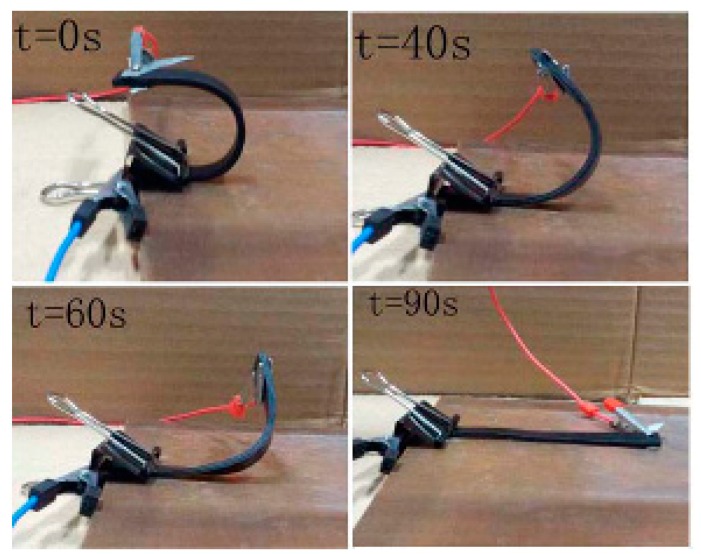
The shape recovery process of the sample with 2.0 wt % graphene under a voltage of 80 V.

The electric field-triggered shape recovery of sample with 2.0 wt % graphene is given in Figure 10. The U shape sample recovers to its initial shape, taking about 90 s under a voltage of 80 V. After approximately 40 s the shape starts to recover. This is because electrically-induced heating generally needs time to build up. Meanwhile, Figure 10 indicates that the shape recovery degree is large, in the range of 40–60 s. This is attributed to the rapid release of the frozen stress. In the final stage of the shape recovery process, the recovery force is gradually reduced to zero. Thus, the shape recovery speed is low.

To investigate the electroactive shape memory property of the graphene/H-EP composites, rectangular samples were tested under 60–160 V voltage. The results show that all of the composite samples can recover to their initial shape under a certain voltage, and the shape recovery ratios are close to 100%. This denotes that the graphene/H-EP composites have excellent electro-active shape memory effect. The shape memory effects of the composites under different voltages are presented in Table 1. As can be seen in the Table 1, the shape recovery time decreases significantly with the increment of the graphene content. Moreover, when the applied voltage increases rapidly, the shape recovery time is greatly reduced.

**Figure 11:**
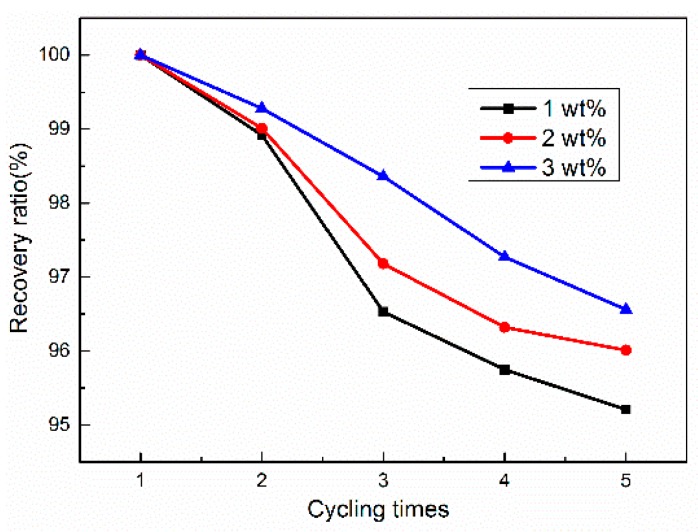
Effects of cycling times on the recovery ratio of graphene/H-EP composites at 120 V.

The creep, plastic deformation, and residual stress will affect the shape memory properties of the composites. Therefore, we tested the shape memory effect of the composites several times to analyze the effect of cycling times on the recovery ratio of graphene/H-EP composites. As is evident from Figure 11, the shape recovery ratios of composites decrease slightly with an increment in cycling time. This could be due to the inevitable plastic deformations and creep caused by the composites. However, it is noted that when the graphene content increases, the shape recovery ratios of the composites increase slightly. This is due to an increase in the stiffness and a decrease in plastic deformation and creep of the composite with increasing graphene content.

## 4. Conclusions

Novel thermo-electrical responsive shape memory graphene/H-EP composites was fabricated and these composites could be developed as MEMS actuation materials for large deformation, multi-stimuli response, and rapid response. Due to the homogeneous dispersion of graphene in the H-EP matrix, the mechanical and electrical conductivity of the composites are improved significantly. The graphene/H-EP composites exhibits excellent electro-active and thermo-active shape memory performance. With the increase of the content of graphene and temperature, the thermo-active shape memory effect is better, and the electro-active shape memory performance of the composites are improved by the increase of the graphene content and applied voltage. Though the cycling times have a disadvantageous effect on shape memory behavior, the shape recovery ratio of the composites are greater than 95%. In their present form, the materials developed are suitable for devices employing low cycling conditions (<5 cycles).

## Figures and Tables

**Table 1:** Recovery time of the composites under different voltage.

Samples	60 V	80 V	100 V	120 V	140 V	160 V
1.0 wt % graphene/H-EP	-	-	-	196 s	138s	87 s
2.0 wt % graphene/H-EP	158 s	90 s	56 s	21 s	5 s	0.8 s
3.0 wt % graphene/H-EP	136 s	72 s	29 s	3 s	0.6 s	0.2 s
